# Valuing patients' experiences of healthcare processes: Towards broader applications of existing methods^☆^

**DOI:** 10.1016/j.socscimed.2014.01.013

**Published:** 2014-04

**Authors:** Mandy Ryan, Philip Kinghorn, Vikki A. Entwistle, Jill J. Francis

**Affiliations:** a Health Economics Research Unit, University of Aberdeen, UK; b Health Economics Unit, University of Birmingham, UK; c Health Services Research Unit, University of Aberdeen, UK; d Social Dimensions of Health Institute, University of Dundee, UK; e School of Health Sciences, City University London, UK

**Keywords:** Quality of healthcare, Patient acceptance of healthcare, Economic evaluation, Process utility, Capabilities approach

## Abstract

Healthcare policy leaders internationally recognise that people's experiences of healthcare delivery are important, and invest significant resources to monitor and improve them. However, the value of particular aspects of experiences of healthcare delivery – relative to each other and to other healthcare outcomes – is unclear.

This paper considers how economic techniques have been and might be used to generate quantitative estimates of the value of particular experiences of healthcare delivery.

A recently published conceptual map of patients' experiences served to guide the scope and focus of the enquiry. The map represented both what health services and staff are like and do and what individual patients can feel like, be and do (while they are using services and subsequently).

We conducted a systematic search for applications of economic techniques to healthcare delivery. We found that these techniques have been quite widely used to estimate the value of features of healthcare systems and processes (e.g. of care delivery by a nurse rather than a doctor, or of a consultation of 10 minutes rather than 15 minutes), but much less to estimate the value of the implications of these features for patients personally.

To inform future research relating to the valuation of experiences of healthcare delivery, we organised a workshop for key stakeholders. Participants undertook and discussed ‘exercises’ that explored the use of different economic techniques to value descriptions of healthcare delivery that linked processes to what patients felt like and were able to be and do. The workshop identified a number of methodological issues that need careful attention, and highlighted some important concerns about the ways in which quantitative estimates of the value of experiences of healthcare delivery might be used. However the workshop confirmed enthusiasm for efforts to attend directly to the implications of healthcare delivery from patients' perspectives, including in terms of their capabilities.

## Highlights

• Significant efforts have been made to *measure* experiences of healthcare delivery.
• Efforts to *value* experiences have focused on healthcare systems and processes.
• The value of their implications for patients personally has been neglected.
• Economic valuation techniques are promising but face challenges in this domain.

## Introduction

Healthcare leaders internationally highlight the importance of patients' experiences of service delivery as well as the prevention and treatment of disease or the improvement of health. The World Health Organization considers ‘responsiveness’ when assessing national health systems (Murray & Evans, 2003), the Institute of Medicine (2001) identifies ‘patient centered care’ as a key dimension of healthcare quality, and many national governments and healthcare organisations include commitments relating to these or similar concepts in their policy and value statements (Department of Health, 2010; Institute for Healthcare Improvement, 2013; NHS Confederation, 2010; The Scottish Government, 2010).

Correspondingly, significant efforts have been made to monitor and improve the quality of people's experiences of healthcare delivery. Extensive surveys of recent service users indicate that, in many countries, people who have accessed care generally rate this as good or satisfactory in most of the domains they are asked about, although there usually continues to be scope for improvement (AHRQ, 2012; Elliott et al., 2010; Richards & Coulter, 2007). Other sources of evidence, including formal complaints, investigative journalism and the official service reviews that these occasionally prompt, indicate that care sometimes falls well short of what is considered acceptable (Francis, 2010, 2013).

There are various reasons for the persistence of shortfalls, and many uncertainties about how best to improve healthcare delivery. Among the issues that warrant attention are the ways in which patients' experiences of healthcare delivery are conceptualised and valued, both ‘officially’ and more informally (even implicitly) by healthcare staff. While most people would agree that positive experiences of healthcare delivery are ‘good things’, in the complex realities of practice, people can characterise, interpret and evaluate particular actions and experiences very differently.

Questions of value have received relatively little attention from researchers when compared to questions of more descriptive measurement in the domains of experiences of healthcare delivery. Significant investments have been made in the development and application of survey methods and instruments for *measuring* (or, more strictly, monitoring indicators of) people's experiences of healthcare. Examples include the NHS Patient Surveys, conducted on behalf of the Care Quality Commission (Picker Institute Europe) and the GP Patient Survey, conducted on behalf of the Department of Health (Ipsos MORI, 2013), both in England, and the Consumer Assessment of Healthcare Providers and Systems (CAHPS) surveys in the USA (AHRQ, 2012). Our concern in this paper is with the relatively neglected possibility of generating quantitative estimates of the *value* that people attach to particular kinds or aspects of experiences of healthcare delivery. We acknowledge that people's evaluations of their healthcare and their healthcare outcomes can influence their responses to questions intended to measure or monitor their experiences or satisfaction (Williams, Coyle, & Healy, 1998), but our interest here is in the possibility of more direct investigations of the values that might influence their evaluations of particular healthcare episodes.

In the next section we outline the conceptualisation of experiences of healthcare delivery that we used to guide our investigation. We then briefly introduce the range of economic techniques that might be used to generate quantitative estimates of the value of experiences of healthcare delivery before turning to the two main components of our investigation: a systematic search that we conducted to identify the aspects of experiences of healthcare delivery that valuation studies have focused on; and a stakeholder workshop conducted to help inform an agenda for future methodological and applied work in this area.

## Which experiences of healthcare delivery matter?

To guide and focus our consideration on valuing experiences of the organisational and interpersonal aspects of healthcare delivery, we used a conceptual map developed to support discussions about the range of such experiences that might matter to people (Entwistle, Firnigl, Ryan, Francis, & Kinghorn, 2012). The map was based on a broad-ranging literature review and a critical interpretive synthesis that highlighted the idea that experiences of healthcare delivery matter because they affect (or represent) aspects of people's wellbeing or the quality of people's lives.

There are several ways of thinking about wellbeing and quality of life. The presentation of patients' experiences of healthcare delivery on the conceptual map that guided this work reflected a ‘capabilities approach’. The capabilities approach treats capabilities for valued functionings as an important informational focus for quality of life assessments (Nussbaum, 2011; Robeyns, 2011; Sen, 1983, 2010). However, while some of the experiences on the conceptual map are presented in terms of capabilities, the insights we generate in this project do not rely on acceptance of the capabilities approach as the best way to assess wellbeing or quality of life.

**Fig. 1:**
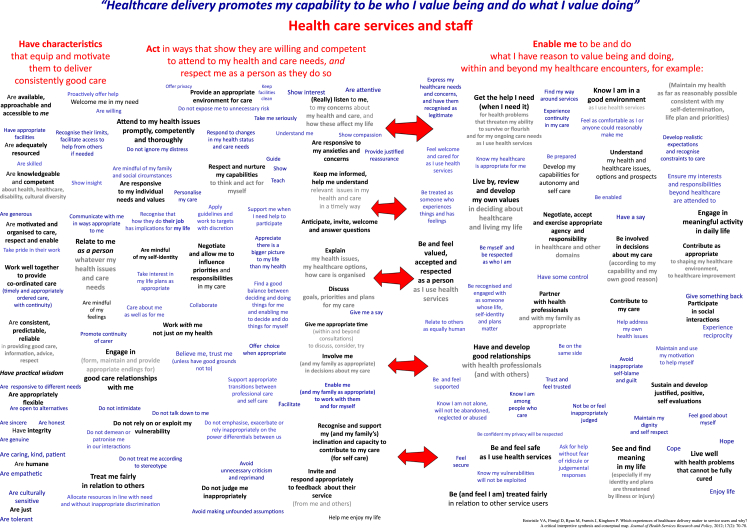
Conceptual map.

The conceptual map, which is reproduced in Fig. 1 below, groups the experience concepts that were identified during the literature review in three main clusters. Moving from left to right across the map, these relate to healthcare services and staff: (1) having characteristics that equip them to deliver consistently good care; (2) acting in ways that show they are willing and competent…; and (3) enabling patients to be and do what they value being and doing … There are multiple conceptual and potentially causal links between the concepts presented under these headings. For example, the notions that healthcare staff ‘explain…’, ‘discuss…’, ‘involve me’ can all be associated with the notions that they enable patients (among other things) to ‘be and feel valued, accepted and respected…’, ‘understand’ and ‘be involved in decisions about my care’.

The conceptual map is messy because it attempts to reflect rather than obscure the complexities of ‘real world’ healthcare delivery. It includes a wide range of experience concepts, and does not assume that these are independent of each other. The generally placed arrows in the middle of the map are intended to recognise and prompt reflection about connections between concepts without implying that there are consistent direct links between specific kinds of experiences. Thus the map aims to show that the implications of what health services and staff are like and do (presented on the left hand side) for aspects of patients' wellbeing or quality of life (presented on the right hand side, under a heading that treats them as capabilities) are in practice complex, non-linear and both person- and context-dependent.

The conceptual map also has several features intended to enhance its usefulness for quality improvement. First, it presents the experiences of healthcare delivery positively (i.e., it is aspirationally worded). Second, it is written from the perspective of an individual patient or service user. This perspective can help emphasise the need for health services and staff to be responsive to individuals: standardised healthcare processes will not necessarily ‘work’ to achieve the aspects of wellbeing or quality of life (framed here as capabilities) on the right hand side of the map for all patients.

However, the features of the map that allow it to reflect the complexities of the real world and to support quality improvement all pose challenges for efforts to value healthcare experiences. The use of economic valuation techniques is made difficult by the broad range of experience concepts and the ambiguities about relationships between them. Also, it is difficult to pinpoint the value of features of healthcare as delivered because the wellbeing or quality of life ‘achievements’ that these might contribute to are also shaped by patients' individual attributes and wider contextual issues. It is to the use of economic evaluation techniques that we now turn.

## Which experiences of healthcare delivery have been valued?

### Methods

We started by identifying economic valuation methods, drawing on and updating a previous systematic review that had looked more broadly at qualitative and quantitative approaches to involving the public in decision making (Ryan et al., 2001). Here we focused on valuation methods that have been used in health economics, or had the potential to be. A summary of all the quantitative methods identified is provided, with references, in Table 1.

Having identified possible economic valuation *methods*, we conducted a systematic search to identify *applications* of these methods to valuing patient experiences of healthcare processes. Detail of the search strategy is provided in online Appendix A. In summary, the search covered MEDLINE, MEDLINE In Process, EMBASE, CINAHL and HMIC databases, and was designed to identify all studies that had applied Willingness-to-Pay (WTP)/Contingent Valuation (CV), Conjoint Analysis (CA), Discrete Choice Experiments (DCE), Best–Worst Scaling (BWS), Swing-Weighting, Measure of Value, Analytical Hierarchical Process (AHP) or Allocation of Points/Budget Pie methods in health-related contexts. Because Standard Gamble (SG) and Time Trade-off (TTO) have been very widely used to value health states, and Person Trade-Off (PTO) to value healthcare interventions, we developed more specific searches for applications of these, incorporating search terms relating to patient experiences of healthcare processes (process utility; process attributes; patient experience; process preferences; waiting time). We also used our own reference lists and emailed experts associated with SG, TTO and PTO, asking if they were aware of any relevant papers, reports or theses not identified by our search. Searches were restricted to documents published in English since 1999. We excluded most studies that had primarily valued health technologies or their attributes (although we included some in which technologies and approaches to healthcare organisation and delivery were closely connected). We also excluded studies that had valued process aspects of healthcare delivery but that had either been conducted in low income countries (where priorities and expectations relating to the processes of healthcare delivery were likely to be significantly different to those in the UK) or had elicited values exclusively from healthcare professionals. We considered the attributes of healthcare delivery that had been valued using economic techniques in relation to the conceptual map (Fig. 1). No quality criteria were applied to inform inclusion decisions or the weight put on study findings, because the focus was on the attributes valued, and their relationship to the experiences represented on the conceptual map. Applying quality criteria would not have been consistent with our research objective.

### Review findings and discussion

3229 records were retrieved from the systematic search. No additional papers were identified from emailing experts, and searches of our reference lists led only to the addition of a PhD thesis, supporting the validity of our systematic search. After de-duplication and screening all titles and abstracts before assessing potentially eligible papers, it was agreed that 89 records valued attributes relating to processes or experiences of healthcare delivery that could somehow be linked to the conceptual map. (The low specificity can be explained by our exclusion criteria, see above). The final 89 included papers are summarised in online Appendix B.

The main finding from this review, summarised in column 4 of Table 1, was that, whilst there is an extensive literature reporting quantitative valuations of structure and process-type attributes of healthcare delivery, most studies have focused on what health services and staff are like or do (or what they ask patients to do). Few attributes have been worded from the perspective of patients and/or oriented to consider explicitly the kinds of impact that healthcare delivery has on them (e.g. how it leaves people feeling, what it enables people to do in consultations or to manage health issues for themselves).

Aspects of service provision that have been valued include (but are not restricted to): location of treatment; travelling time/distance to clinic/appointment; cost of travelling; waiting time for an appointment/on a waiting list; whether the member of staff seen is familiar/continuity of staff seen; length of consultation; privacy (when receiving test results). Examples of the use of DCE include the valuation of: location of care, waiting time, travel time, specialized experienced provider, and staff continuity, in the context of public preferences for surgical care provision (Schwappach & Strasmann, 2007); and an investigation of the relative importance of factors influencing patient choice when booking general practice appointments, which included the following attributes: day of appointment, type of professional person seen, time of day of appointment, and length of appointment (Gerard, Salisbury, Street, Pope, & Baxter, 2008). Willingness to pay has been used to value reduced waiting time for cataract surgery (Bishai & Chu Lang, 2000) and to examine the relative preference of patients with chronic heart failure and hypertension to be examined by their doctor at home via telemedicine, rather than attend the clinic (Bradford, 2005). Best–Worst Scaling has been used to value different levels of waiting time, specialist expertise, convenience of appointment and thoroughness of consultation in the provision of a dermatology consultation (Coast et al., 2006). Standard gamble has been used to value hospital versus GP-managed care in patients with atrial fibrillation (Robinson, Thomson, Parkin, Sudlow, & Eccles, 2001) and the chained standard gamble approach has been used to capture process utility in the context of conservative versus aggressive follow-up of mildly abnormal pap smears (Birch, Melnikow, & Kuppermann, 2003).

Some aspects of interactions with staff have also been valued, including: attitudes of healthcare staff; patient involvement in decision making; and amount of information provided (and how well it is explained). A limited number of studies have characterised at least some of these aspects of interaction, at least implicitly, from the perspective of patients. For example, in a study concerned with patient involvement in primary care consultations, attributes valued included: whether the doctor listens (Doctor does not seem to listen/Doctor seems to listen) and how easily the information is understood (Difficult to understand/Easy to understand) (Longo et al., 2006). A study concerned with the impact of non-health attributes of care on patients' choice of GP considered the value of ‘the doctor treats you with dignity’, ‘the doctor recognises your pain/stress’, ‘the doctor takes notice of what you say or do about your health’, ‘the doctor reassures you’, ‘the doctor is trustworthy’, and ‘the doctor gives you information’ (Haas, Sharma, & Stano, 2005).

Few studies have attempted to value *directly* the kinds of personally experienced aspects of wellbeing or quality of life that healthcare delivery can impact on. Ambiguities in wording can sometimes support an interpretation that attributes do reflect patients' perspectives. For example, ‘the doctor reassures you’ could be interpreted with an emphasis on either what the doctor is perceived to have tried to do or on the achievement of the individual being reassured. And valuations of some of the experiences represented on the right hand side of the map may have been *indirectly* reflected in the values attached to the kinds of experiences represented on the left hand side of the map. For example, when considering the value of a 10-minute versus 20-minute wait, respondents might consider the implications of this for aspects of their wellbeing such as capabilities to: ‘get the help I need when I need it’; ‘be and feel I am treated fairly in relation to other service users’; and ‘be and feel respected as a person’. However, the values of the implications that features of healthcare delivery generate for patients (the values of ‘what it is like for me’) have not been directly and explicitly examined. Further, the relationships between many of the attributes that have been valued and the experiences represented on the map are unlikely to be consistent across individuals. This is because the particular features of healthcare provision that are described in terms of objectively categorised units (professional qualifications, time periods etc.) will have different implications (e.g. will convert into different capabilities) for different people.

Some studies have valued what patients feel like or are able to do, but they have tended to do so without regard for whether and how these experiences might have been supported or undermined by healthcare services or staff. For example, a DCE to investigate what outcomes are important to people with long-term conditions incorporated a self-efficacy attribute with three levels: ‘totally confident in ability to manage condition’, ‘moderately confident in ability to manage condition’ and ‘not at all confident in ability to manage condition’ (Richardson et al., 2009).

**Fig. 2:**
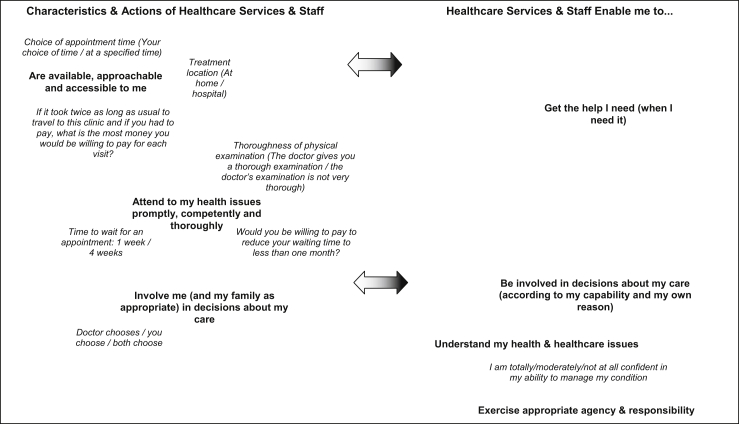
Aspects of experience from the conceptual map with related attributes identified in the second systematic search.

Fig. 2 illustrates how some of the attributes that economists have valued (presented in italics) might be related to a selection of three linked aspects of experience from the left hand side and four from the right hand side of the conceptual map (presented in bold). The absence of empirically studied attributes on the right-hand-side of Fig. 2 is striking.

## Assessing the feasibility of valuation methods for valuing patient experiences

It might be thought that valuations of objectively measurable units of healthcare provision and processes can suffice to inform policy and service planning IF they can adequately reflect valuations of the consequences of those units for patients. But respondents to valuation exercises will often not know those consequences, and the consequences are likely to be different for, and differently valued by, different patients. We therefore suggest that empirical investigations of the value people attach to the more outcome-like impacts of healthcare delivery that are represented on the right hand side of the conceptual map are warranted – at least to establish how they relate to investigations of the value people attach to healthcare structures and processes.

The question then emerges of whether and how the valuation techniques identified in Table 1 could be used to elicit values for attributes on the right on the conceptual map. We used a stakeholder consultation workshop to consider this.

### Developing scenarios for valuation

To assess the feasibility of using economic methods to value what patients experience as a result of healthcare delivery, we developed one plausible scenario, with a manageable number of attributes (five) and levels (one positive and one negative for each attribute), reflecting a range of the types of experience represented on the conceptual map.

**Fig. 3:**
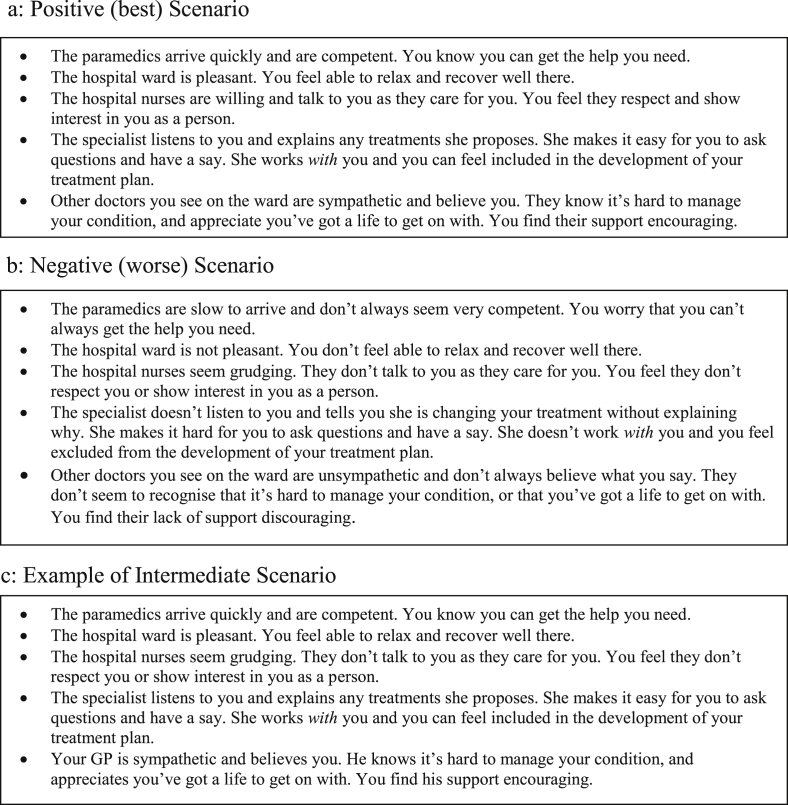
Examples of scenarios derived from the conceptual map and used in the workshop valuation tasks.

The scenario invited people to imagine they had a (generic) long-term health condition requiring daily medication, regular consultations with healthcare staff, and occasional unplanned hospitalisations for short intervals. The best positive and worst negative versions of the scenario are presented in Fig. 3 (Fig. 3a and b) together with an intermediate version (Fig. 3c). Concepts from both sides of the conceptual map were combined into single attributes to ‘link’ patients' personal experiences to the healthcare they had received. For example, the attribute “The hospital ward is pleasant. You feel able to relax and recover well there” relates to (from Fig. 1) “provide an appropriate environment for care” (left hand side), and “know I am in a good environment…” and “feel… comfortable…” (right hand side).

We referred to different healthcare providers for each attribute to help ensure plausibility when both positive and negative attribute levels occurred within a scenario (it might be hard to believe the same person would simultaneously provide some combinations of care).

### Workshop methods

The feasibility of using the methods to value patient experiences of healthcare processes was explored at a stakeholder consultation workshop, using the scenario described above and questions/exercises based on each of the valuation methods. The workshop brought together economists (5), policy makers from Department of Health (England) and the Scottish Government (11), academics from several health-related disciplines (4), and voluntary sector patient advocates (2). Participants were divided into small groups (of around five people with different backgrounds) and given a workshop pack. The pack contained background information, informing participants that policy makers are placing increasing importance on the patient's experience of receiving healthcare, that decisions have to be made about priorities and that we would be interested in how much value they place on having a good experience of healthcare. It also contained the valuation tasks, in the format in which they would be presented to an “ordinary” respondent, together with guidance on how to complete the tasks. Members of the project team introduced the project (MR, VE and PK) and gave short introductions to the economic valuation methods in four sessions (MR, PK) (see Appendix).

Not all the methods listed in Table 1 were included in the workshop due to time constraints. Some methods were excluded in part because they had had very limited application in health economics since the Ryan et al. (2001) report which recommended their application be explored. These included AHP/ANP, Measure of Value and allocation of points. We employed two variants of Conjoint Analysis (ranking and rating), one variant of VAS (Swing-Weighting) and one variant of SG (perhaps more accurately referred to as certainty equivalent). Participants were encouraged to complete the tasks, annotate their pack with comments on how they found the tasks, and discuss their views of the tasks within their group. Discussion was not audio-recorded, but a member of the research team observed each group and made notes, and annotated information packs were collected at the end of the workshop. Several participants also sent retrospective reflections by email. The research team met after the workshop to reflect on key points discussed/raised. The insights presented below are thus a combination of quotes taken from workshop participants (identified in quotation marks and by Participant number), feedback from participants post workshop and the reflections of the research team.

### Insights generated from the workshop

When working through techniques involving monetary measures (See Appendix – Sessions 1 and 2), participants did not object in principle to being asked about paying for an improved patient experience, but some thought it implausible that paying more would ensure an improved experience. Indeed, policy makers questioned whether poor care resulted from a lack of funds or, alternatively, from poor management.

*“Challenge is that cost is not necessarily what relates to poor care. …I don't necessarily believe that poor care (as described) is because of insufficient funds.*” [Participant 4]

Regarding the techniques involving non-monetary measures (see Appendix – Sessions 3 and 4), participants expressed no objections to the methods presented. Feedback to the standard gamble task was generally positive, and one participant noted it was “*relatively easy*.” Some respondents discussed the possibility that they would rather die sooner than put up with ‘bullying’ nurses if they were dependent on nursing care, which suggested a time trade off method could be applicable in some scenarios. When attempting the person trade off exercise, participants were confused by what would happen to the patients who were not treated:

*“I don't think I can answer this without the counter. What happens to the people who aren't treated? Paramedics don't come?*” [Participant 1]

Participants also wanted to know how many patients needed treatment (i.e. how many, from the total number needing treatment, would be refused it):

*“Difficult to conceptualise, particularly person trade-off –how many people need treatment?*” [Participant 6]

In addition to specific comments regarding the valuation techniques, a number of general issues arose in the workshop. There was discussion around the importance of considering interactions between attributes e.g. with an effective GP you might expect fewer hospital visits, so the attributes relating to hospital care wouldn't matter so much. We therefore suggest that future work attempting to value patient experiences of healthcare delivery should explore inclusion of interaction terms. (This is consistent with Louviere and Lancsar's (2009) suggestion that identification of which model effects can be estimated is a crucial area for future research relating to discrete choice experiments. However, we suggest it will be important for *all* economic valuation methods.)

Some workshop participants indicated concern that they did not have more information about the impact of the care described in the scenarios on health outcomes. Some felt that, had health attributes been included in the scenarios, then the idea of trading life years or risk of death would have been more acceptable.

Unless patient experiences of healthcare processes and health attributes are valued on the same scale, it will not be possible to quantify trade-offs between them. However, a strong view voiced by a number of participants was that some of the experiences on the conceptual map relate to basic human rights and should not be traded. This view reflects current guidance from the Department of Health (2008, p6), which highlights that ‘*Human rights are not an “add-on”, they are an inherent part of care’* and affirms the ‘FREDA’ values of Fairness, Respect, Equality, Dignity and Autonomy. However, resources are likely to be needed to ensure that the process of care does, in practice, respect these values.

In summary, participants at the workshop saw the potential for both monetary and non-monetary valuation methods to value the kinds of experience represented on the conceptual map. Methods that have not previously been widely used to value process-type attributes, including standard gamble and time trade off techniques, were also seen as potentially useful. Future work should also explore if these findings hold for the general population.

As noted above, time constraints meant that not all the methods listed in Table 1 were included. Future work could usefully explore the application of allocation of points, analytical hierarchical process and measure of value in health economics generally as well as in the valuation of patients' experiences more particularly.

The scenario that we used in our initial exploration of the potential of a diverse range of economic methods to value implications of healthcare delivery for patients characterised these implications in terms of capabilities. Although we believe that the adoption of insights from the capabilities approach may have several advantages for efforts to improve the quality of healthcare (Entwistle et al., 2012; Entwistle & Watt, 2013), we do not wish to suggest that all future work on patients' experiences of healthcare delivery should be framed within it.

We identified several methodological issues before, during and after the workshop.

First, our scenario development was not systematic. An important line for future research is to use frameworks for reducing the number of concepts on the map to a smaller number for defining attributes, levels and scenarios. Possible approaches include Q-methodology (Baker, 2006; Baker, Thompson, & Mannion, 2006) and Rasch Analysis (Ryser, Wright, Aeschlimann, Mariacher-Gehler, & Stucki, 1999; Tennant, McKenna, & Hagell, 2004).

Second, in our scenario development we used a *direct* approach to value experiences represented on the right hand side of the conceptual map. This requires respondents to regard descriptions of “what it's like for patients” (in our case, what capabilities patients have) as plausible. It might be that individuals are not familiar with the experiences (have not had the capabilities) that are being valued, and might not regard them as plausible. This would clearly threaten the validity of the valuation exercise.

An alternative approach to valuation would be to value the more outcome-like patient experiences *indirectly* in multi-component studies. For example, valuation tasks that focus on healthcare structures and processes (the kind of tasks that have most often been used to date) could be complemented by collecting information about what implications (e.g. capabilities) respondents associated particular processes with. The value of such implications (e.g. capabilities) could then be estimated indirectly, using regression techniques. This kind of approach has been used before to value internal states, although the implication of interest was singular and fixed for all respondents. Ryan (1998) investigated the value of the internal state of ‘*knowing you have done everything possible to have a child*’ following fertility treatment. Rather than ask individuals to assume this, which may be unrealistic and therefore inappropriate for some individuals, she asked respondents to state their level of agreement with that statement, “I know I have done everything possible to have a child” and then used regression techniques to relate agreement levels to willingness to pay for infertility treatment.

## Conclusions

In a linked series of studies, we have: identified what experiences of healthcare delivery matter to patients (Entwistle et al., 2012); identified methods used by economists to value experiences of healthcare processes; systematically searched for previous applications of these methods and found that they have focused on a limited range of experiences, usually characterised in terms of the structures and processes of healthcare; and initiated investigations of the feasibility of using available valuation methods to value a broader range of experiences, including the implications of healthcare delivery for individuals (including their capabilities). A large number of structure- and process-type attributes have been valued by health economists, using a range of valuation methods. These can provide useful information for policy makers. However, when considered in the light of the conceptual map of experiences of healthcare processes identified as mattering to patients, the range and characterisation of domains that have been valued seems limited. In particular, very little work has valued the impact of healthcare processes on how individuals are enabled in domains that matter to them.

Valuation of the broader, including the more ultimate, kinds of experience of healthcare delivery that are represented on the conceptual map will be challenging. The concepts on the conceptual map are related to each other in a variety of ways and economic techniques can feasibly generate estimates of the value of only a limited number of domains at one time. Methodologically, it is not yet clear how best to go about developing a manageable set of attribute descriptors for valuation, or whether the implications of healthcare delivery in terms of patients' feelings, capabilities etc. should be valued *directly* (as in the approaches explored in our Workshop) or *indirectly* (by collecting additional information alongside valuation of processes). Methodological work will also be needed to explore and deal with interactions between patient experience domains and health outcomes in the valuation tasks.

The potential to generate quantitative estimates of the value of patients' experiences of healthcare delivery also raises ethical questions. The achievement of at least minimum threshold for some of the experiences represented on the conceptual map may be regarded as non-negotiable, a matter of human rights. This raises questions about the appropriateness of including some kinds of experiences in trade-off exercises, and about the uses that can legitimately be made of the outputs of economic valuation exercises.

Conceptual, methodological and ethical challenges clearly lie ahead, but efforts to improve understanding of the values that can be attached to patients' experiences of healthcare delivery are needed to complement understandings of the values that can be attached to different health states, and thus to inform policy and service development.

## Figures and Tables

**Table 1:** Methods used by economists, and their application to valuing patients' experiences of the processes of healthcare.

Valuation method	Variations	Key references/Review papers explaining methods	Examples of attributes valued and relationship to conceptual map
Allocation of Points	Budget Pie	Hoinville, (1996)	None
Analytic Hierarchical Process	Analytic Network Process^a^	Dolan, Isselhardt, and Cappuccio (1989) Saaty (1999)	None
Best–Worst Scaling^a^ (BWS)	Best–Worst Attribute Scaling^a^ Best–Worst Object Scaling^a^ Best–Worst DCE^a^	Flynn, Louviere, Peters, and Coast (2007) Lee, Soutar, and Louviere (2008) Lancsar and Louviere (2009)	Small but growing literature used to directly value both aspects of the conceptual map from the LHS (Coast et al., 2006; Flynn, Louviere, Peters, & Coast, 2008) and within the framework of the Capabilities Approach (Coast, Smith, & Lorgelly, 2008; Coast, Flynn, Sutton, et al., 2008) *Directly* valued aspects on both the LHS and RHS of the conceptual map.
Contingent Valuation (CV)	Open-ended Payment card Dichotomous choice – Single and double bounded, random sorting	Donaldson (2011)	Extensive literature directly valuing LHS of conceptual map, but focusing on process descriptors (e.g. location of treatment; travelling time/distance to clinic/appointment; waiting time for an appointment/on a waiting list; whether you see a member of staff who is familiar to you/continuity of staff seen; distance to treatment centre; cost of travelling; length of consultation; privacy (when receiving test results) and *interactions with staff* (attitudes of healthcare staff; patient involvement in decision making; level of information provided and how well it is explained); and skill level of the healthcare staff). Limited application to value LHS domains from perspective of individual. Not used to directly value aspects of the RHS of the conceptual map.
Discrete Choice Experiments (DCE)		de Bekker-Grob, Ryan, and Gerard (2012)	Same as Contingent Valuation above. However, limited studies identified valuing RHS of map (Longo et al., 2006; Richardson et al., 2009).
Measure of Value		Churchman and Ackoff (1954)	None
Person Trade-Off (PTO)		Green, (2001)	None
Rating Scale	Conjoint analysis (CA) rating scales Visual Analogue Scale (VAS) Swing-Weighting Method^a^	Ryan et al. (2001) Parkin, (2006) von Winterfeldt and Edwards (1986)	CA rating scales focused on LHS of conceptual map (Ryan & Farrar, 2000; Ryan, McIntosh, & Shackley, 1998), with a focus on process descriptors. VAS – None Swing weighting – one study used within Capabilities Framework (Kinghorn, 2010).
Standard Gamble (SG)		Gafni, (1994)	Five papers identified valuing directly process type factors, focusing on descriptors of the process (Birch et al., 2003; Robinson et al., 2001). Not used to value aspects from RHS of the conceptual map.
Time Trade-Off (TTO)		Torrance, Thomas, and Sackett (1972) Torrance, G. (1986)	Three papers used TTO to value process type factors from the LHS of conceptual map, focusing on descriptors of the process, E.g. McNamee and Seymour (2008). Not used to value aspects from RHS of the conceptual map.

a New methods since Ryan et al. (2001) review.

## Appendix Workshop exploring feasibility of different instruments for valuing patient experiences of the process of healthcare

*Session 1* – Direct Monetary Measures (WTP/CV); *Session 2* – Indirect Monetary Measures (DCE, CA ranking and CA rating all including a price proxy); *Session 3* – Nonmonetary Measures (CA and DCE without price proxy; swing-weighting and BWS); *Session 4* – Nonmonetary Measures (SG, TTO and PTO).

In *Session 1* (CV/WTP) participants were asked to imagine they lived in a country like the United States where healthcare is funded through private insurance. An initial scenario was presented in which the patient experience was relatively poor. Respondents were asked how much extra they would be willing to pay in additional monthly insurance for an improvement in the levels of some attributes.

In *Session 2*, using the DCE, participants were asked to choose one insurance package from a choice of two, differing in the level of attributes (patient experiences). For the ranking exercise participants were asked to rank three insurances packages and for the rating exercise to rate two packages on a scale of one to 10, where one was ‘*worst imaginable package*’ and 10 ‘*best imaginable package*’. All exercises included a cost attribute (monthly insurance premium).

*Session 3* repeated these three methods without a cost attribute, and also included a swing-weighting and BWS exercise. The swing-weighting method followed Peacock, Richardson, Carter, and Edwards (2007) and Kinghorn (2010) and the BWS followed Coast, Flynn, Natarajan, et al. (2008).

*In Session 4* SG participants were asked to imagine they required five days in hospital as part of their treatment. Their GP could organise treatment in Hospital A with certainty, with a relatively negative patient experience. They could refuse Hospital A, and be treated at one of two alternative hospitals, X or Y (depending on availability of beds). If they refuse treatment at Hospital A, they must accept treatment at the alternative. There was a 95% probability of being sent to Hospital X, with an entirely positive patient experience and a 5% chance of being sent to Hospital Y, with an entirely negative patient experience. Participants were asked to choose between sure treatment at Hospital A, or the gamble between Hospitals X and Y. For the TTO participants were asked if they would prefer to live for *t* years while receiving a poor experience of care, or live for some shorter duration while receiving an entirely positive patient experience (or indifferent between options). For the PTO participants were asked their preference over treating *x* patients with a relatively poor healthcare experience, or treating a lower number of patients with a more positive healthcare experience; it was stated that in this latter case some patients would not receive treatment.

## References

[bib1] AHRQ (2012). National disparities report 2011.

[bib2] Baker R. (2006). Economic rationality and health and lifestyle choices for people with diabetes. Social Science & Medicine.

[bib3] Baker R., Thompson C., Mannion R. (2006). Q methodology in health economics. Journal of Health Services Research & Policy.

[bib46] de Bekker-Grob E., Ryan M., Gerard K. (2012). Discrete choice experiments in health economics: a review of the literature. Health Economics.

[bib4] Birch S., Melnikow J., Kuppermann M. (2003). Conservative versus aggressive follow up of mildly abnormal Pap smears: testing for process utility. Health Economics.

[bib5] Bishai D.M., Chu Lang H. (2000). The willingness to pay for wait reduction: the disutility of queues for cataract surgery in Canada, Denmark, and Spain. Journal of Health Economics.

[bib6] Bradford W.D., Kleit A., Krousel-Wood M.A., Re R.M. (2005). Comparing willingness to pay for telemedicine across a chronic heart failure and hypertension population. Telemedicine and e-Health.

[bib47] Churchman C.W., Ackoff R.L. (1954). An approximate measure of value. Journal of the Operations Research Society of America.

[bib48] Coast J., Flynn T., Natarajan L., Sproston K., Lewis J., Louviere J.J. (2008). Valuing the ICECAP capability index for older people. Social Science & Medicine.

[bib49] Coast J., Flynn T., Sutton E., Al-Janabi H., Vosper J., Lavender S. (2008). Investigating choice experiments for preferences of older people (ICEPOP): evaluating spaces in health economics. Journal of Health Services Research Policy.

[bib50] Coast J., Smith R.D., Lorgelly P. (2008). Should the capability approach be applied in health economics?. Health Economics.

[bib7] Coast J., Salisbury C., de Berker D., Noble S., Horrocks S., Peters T. (2006). Preferences for aspects of a dermatology consultation. British Journal of Dermatology.

[bib8] Department of Health (2008). Human rights in healthcare: A short introduction.

[bib9] Department of Health (2010). Equity and excellence: Liberating the NHS.

[bib51] Dolan J.G., Isselhardt B.J., Cappuccio J.D. (1989). The analytical hierarchy process in medical decision making: a tutorial. Medical Decision Making.

[bib10] Donaldson C. (2011). Willingness to pay and publicly funded health care: contradictions in terms?. Office of health economics seminar briefing note November.

[bib11] Elliott M.N., Lehrman W.G., Goldstein E.H., Giordano L.A., Beckett M.K., Cohea C.W. (2010). Hospital survey shows improvements in patient experience. Health Affairs.

[bib12] Entwistle V., Firnigl D., Ryan M., Francis J., Kinghorn P. (2012). Which experiences of health care delivery matter to service users and why? A critical interpretive synthesis and conceptual map. Journal of Health Services Research & Policy.

[bib13] Entwistle V., Watt I.S. (2013). Treating patients as persons: a capabilities approach to support delivery of person-centred care. American Journal of Bioethics.

[bib52] Flynn T., Louviere J.J., Peters T., Coast J. (2007). Best-worst scaling: what it can do for health care research and how to do it. Journal of Health Economics.

[bib15] Flynn T., Louviere J.J., Peters T., Coast J. (2008). Estimating preferences for a dermatology consultation using Best–Worst Scaling: comparison of various methods of analysis. BMC Medical Research Methodology.

[bib16] Francis R. (2010). Independent inquiry into care provided by Mid Staffordshire NHS Foundation Trust January 2005–March 2009.

[bib17] Francis R. (2013). The Mid Staffordshire NHS Foundation Trust public enquiry.

[bib53] Gafni A. (1994). The standard gamble method: what is being measured and how it is interpreted. Health Services Research.

[bib18] Gerard K., Salisbury C., Street D.J., Pope C., Baxter H. (2008). Is fast access to general practice all that should matter? A discrete choice experiment of patients' preferences. Journal of Health Services Research & Policy.

[bib54] Green C. (2001). On the societal value of health care: what do we know about the Person trade-off technique?. Health Economics.

[bib19] Haas M., Sharma R., Stano M. (2005). Cost-effectiveness of medical and chiropractic care for acute and chronic low back pain. Journal of Manipulative and Physiological Therapeutics.

[bib55] Hoinville G. (1996). Evaluating community preferences. Journal of the Market Research Society.

[bib20] Institute for Healthcare Improvement, http://www.ihi.org/about/pages/default.aspx Accessed 10.11.13.

[bib21] Institute of Medicine, Committee on Quality of Health Care in America (2001). Crossing the quality chasm.

[bib22] Ipsos MORI. *The GP Patient Survey*. http://www.gp-patient.co.uk/ Accessed 21.02.13.

[bib23] Kinghorn P. (2010). Developing a capability approach to measure and value quality of life: An application to chronic pain.

[bib56] Lancsar E., Louviere J.J. (2009). Estimating individual level discrete choice models and welfare measures using best worst choice experiments. Health Economists' Study Group Meeting.

[bib57] Lee J.A., Soutar G., Louviere J.J. (2008). The best-worst scaling approach: an alternative to Schwartz's value survey. Journal of Personality Assessment.

[bib24] Longo M.F., Cohen D.R., Hood K., Edwards A., Robling M., Elwyn G. (2006). Involving patients in primary care consultations: assessing preferences using discrete choice experiments. British Journal of General Practice.

[bib25] Louviere J.J., Lancsar E. (2009). Choice experiments in health: the good, the bad, the ugly and toward a brighter future. Health Economics, Policy & Law.

[bib58] McNamee P., Seymour J. (2008). Incorporation of process preferences within the QALY framework: a study of alternative methods. Medical Decision Making.

[bib26] Murray C., Evans D.B. (2003). Health System Performance Assessment: Debate, methods and empiricism.

[bib27] NHS Confederation (2010). Feeling better? Improving patient experience in hospital. http://www.nhsconfed.org/publications/reports/pages/feeling-better-improving-patient-experience-in-hospital.aspx.

[bib28] Nussbaum M. (2011). Creating capabilities: The human development approach.

[bib59] Parkin D. (2006). Is there a case for using visual analogue scale valuations in cost-utility analysis?. Health Economics.

[bib29] Peacock S.J., Richardson J., Carter R., Edwards D. (2007). Priority setting in health care using multi-attribute utility theory and programme budgeting and marginal analysis. Social Science & Medicine.

[bib30] Picker Institute Europe. *NHS Surveys: Focused on patients' experience*. http://www.nhssurveys.org/ Accessed 21.02.13.

[bib31] Richards N., Coulter A. (2007). Is the NHS becoming more patient-centred? Trends from the national surveys of NHS patients in England 2002–2007.

[bib32] Richardson G., Bojke C., Kennedy A., Reeves D., Bower P., Lee V. (2009). What outcomes are important to patients with long term conditions? A discrete choice experiment. Value in Health.

[bib33] Robeyns I., Zalta E. (2011). The capability approach. The Stanford encyclopedia of philosophy.

[bib34] Robinson A., Thomson R., Parkin D., Sudlow M., Eccles M. (2001). How patients with atrial fibrillation value different health outcomes: a standard gamble study. Journal of Health Services Research & Policy.

[bib35] Ryan M. (1998). Valuing psychological factors in the provision of assisted reproductive techniques using the economic instrument of willingness to pay. Journal of Economic Psychology.

[bib36] Ryan M., Farrar S. (2000). Using conjoint analysis to elicit preferences for health care. BMJ.

[bib37] Ryan M., McIntosh E., Shackley P. (1998). Using conjoint analysis to elicit the views of health service users: an application to the patient health care. Health Expectations.

[bib38] Ryan M., Scott D.A., Reeves C., Bate A., van Teijlingen E., Russell E.M. (2001). Eliciting public preferences for healthcare: a systematic review of techniques. Health Technology Assessment.

[bib39] Ryser L., Wright B.D., Aeschlimann A., Mariacher-Gehler S., Stucki G. (1999). A new look at the Western Ontario and McMaster Universities Osteoarthritis Index using Rasch analysis. Arthritis Care & Research.

[bib60] Saaty T.L. (1999). Fundamentals of the analytic network process. International Symposium on the Analytic Hierachical Process.

[bib40] Schwappach D., Strasmann T.J. (2007). Does location matter? A study of the public's preferences for surgical care provision. Journal of Evaluation in Clinical Practice.

[bib41] Sen A. (1983). Poor, relatively speaking. Oxford Economic Papers.

[bib42] Sen A. (2010). The idea of justice.

[bib43] Tennant A., McKenna S.P., Hagell P. (2004). Application of Rasch analysis in the development and application of quality of life instruments. Value in Health.

[bib44] The Scottish Government (2010). The healthcare quality strategy for Scotland.

[bib61] Torrance G., Thomas W., Sackett D. (1972). A utility maximisation model for evaluation of health care programs. Health Services Research.

[bib45] Williams B., Coyle J., Healy D. (1998). The meaning of patient satisfaction: an explanation of high reported levels. Social Science & Medicine.

[bib62] von Winterfeldt D., Edwards W. (1986). Decision analysis and behavioural research.

