# Supplementary material for: Valuing patients' experiences of healthcare processes: Towards broader applications of existing methods
Source: Soc Sci Med. 2014 Apr;106(100):194–203. doi: 10.1016/j.socscimed.2014.01.013 (PMC3988932; doi:10.1016/j.socscimed.2014.01.013)
Supplement: Supplementary file 1 [file mmc1.docx]

**Online Appendices:**

**Appendix A: Search Terms & Results**

Sensitive searches were developed to retrieve papers that had used the techniques identified from Stage 1. Scoping searches had shown that there was a large body of literature employing the more established techniques (Contingent Valuation (CV), Willingness to pay (WTP), Discrete Choice Experiments (DCEs) and Conjoint Analysis (CA)) so for these techniques, searches were restricted to the healthcare databases (MEDLINE, MEDLINE In Process, EMBASE, CINAHL and HMIC). For the other identified techniques, all the listed healthcare databases as well as those used in Stage 1 were searched. All searches were for documents published from 1999 onwards in the English language.

| Database | Number of reports retrieved |
| --- | --- |
| MEDLINE, MEDLINE In - Process EMBASE multifile search | 2212 |
| CINAHL | 799 |
| HMIC | 218 |
| TOTAL | 3229 |

In total, 3229 records were retrieved but this total was reduced to 2463 after de-duplication.

***Database: EMBASE (1999 - 2010 Week 21, MEDLINE (1999 - May Week 3 2010, MEDLINE(R) In-Process 28^th^ May 2010)***

1 (contingent adj2 valu$).tw.

2 willingness to pay.tw.

3 wtp.tw

4 discrete choice$.tw.

5 (conjoint adj2 analys?s).tw.

6 analytic$ hierarch$.tw.

7 best worst.tw.

8 (budget$ adj2 pie$).tw.

9 (allocat$ adj2 point$).tw

10 (priorit$ adj2 evaluat$).tw.

11 swing weight$.tw

12 measure of value.tw.

13 standard gamble$.tw

14 trade off.tw.

15 (tto or pto).tw.

16 (process$ adj3 (attribut$ or utilit$ or preference$)).tw.

17 (patient$ adj3 experience$).tw.

18 (wait$ adj3 time).tw

19 16 or 17 or 18

20 (13 or 14 or 15) and 19

21 or/1-12, 20

22 limit 21 to english language

23 limit 22 to yr="1999 -Current"

24 remove duplicates from 23

***CINAHL (1999- May 2010)***

S1 TX contingent w2 valu* or TX willingness to pay or TX wtp

S2 TX discrete choice* or TX conjoint w2 analysis or TX conjoint w2 analyses

S3 TX analytic* hierarch* or TX best worst or TX budget* w2 pie*

S4 TX allocat* w2 point* or TX priorit* w2 evaluat*

S5 TX swing weight* or TX “measure of value”

S6 TX standard gamble* or TX trade off or TX tto or TX pto

S7 TX process* w3 attribut* or TX process* utilit* or TX process* preference*

S8 TX patient* w3 experience* or TX wait* w3 time

S9 S7 or S8

S10 S6 and S9

S11 S1 or S2 or S3 or S4 or S5 or S10

S12 S11Limiters - Language: English

***Health Management Information Consortium (HMIC) 1999 - April 2010***

1 (contingent adj2 valu$).tw.

2 willingness to pay.tw.

3 wtp.tw.

4 discrete choice$.tw.

5 (conjoint adj2 analys?s).tw.

6 analytic$ hierarch$.tw.

7 best worst.tw.

8 (budget$ adj2 pie$).tw.

9 (allocat$ adj2 point$).tw.

10 (priorit$ adj2 evaluat$).tw

11 swing weight$.tw

12 measure of value.tw.

13 standard gamble$.tw

14 trade off.tw.

15 (tto or pto).tw.

16 (process$ adj3 (attribut$ or utilit$ or preference$)).tw.

17 (patient$ adj3 experience$).tw.

18 (wait$ adj3 time).tw

19 16 or 17 or 18

20 (13 or 14 or 15) and 19

21 or/1-12, 20

22 limit 21 to yr="1999 -Current"

***EconLit (1999 – May 2010)***

S1 TX analytic* hierarch*

S2 TX "best worst"

S3 TX budget* W2 pie*

S4 TX allocat* W2 point*

S5 TX priorit* N2 evaluat*

S6 TX economic* W2 happiness

S7 TX subjective N2 wellbeing or TX subjective N2 well being

S8 S1 or S2 or S3 or S4 or S5 or S6 or S7

S9 S1 or S2 or S3 or S4 or S5 or S6 or S7 Limiters - English Only

***RePEC 3^rd^ June 2010***

“Analytic hierarchy”

OR

“best worst”

OR

“budget pie” | “budgetary pie” | “budgeting pie”

OR

“allocate points” | allocation of points”

OR

"priority evaluator" | "priorities evaluator" | "priority evaluation" | "priorities evaluation" | "evaluation of priorities"

OR

"economics of happiness"

OR

"subjective wellbeing" | "subjective well being"

***International Bibliography of the Social Sciences (1999 - May 2010)***

S1 TX analytic* hierarch*

S2 TX "best worst"

S3 TX budget* W2 pie*

S4 TX allocat* W2 point*

S5 TX priorit* N2 evaluat*

S6 TX economic* W2 happiness

S7 TX subjective N2 wellbeing or TX subjective N2 well being

S8 S1 or S2 or S3 or S4 or S5 or S6 or S7

S9 S1 or S2 or S3 or S4 or S5 or S6 or S7 Limiters - English Only

***ERIC (1999 - April 2010)***

1 analytic$ hierarch$.tw.

2 best worst.tw.

3 (budget$ adj2 pie$).tw.

4 (allocat$ adj2 point$).tw

5 (priorit$ adj2 evaluat$).tw

6 (economic$ adj2 happiness).tw.

7 (subjective adj2 (wellbeing or well being)).tw.

8 or/1-7

9 limit 8 to yr="1999 -Current"

10 limit 9 to english language

***PsycINFO (1999 - May Week 4 2010)***

1 analytic$ hierarch$.tw.

2 best worst.tw.

3 (budget$ adj2 pie$).tw.

4 (allocat$ adj2 point$).tw

5 (priorit$ adj2 evaluat$).tw

6 (economic$ adj2 happiness).tw.

7 (subjective adj2 (wellbeing or well being)).tw.

8 7 and (scale? or scaling).tw,hw.

9 7 and (rate or rating).tw,hw.

10 or/1-6,8,9

11 limit 10 to yr="1999 -Current"

12 limit 11 to english language

***Environmental Science and Pollution Management (1999 – May 2010)***

(analytic* hierarch*) or (best worst) or (budget* within 2 pie*) or (allocat* within 2 point*) or (priorit* within 2 evaluat*) or (economic* within 2 happiness)

**Appendix B: Studies including attributes relating to patient experience**

| **Author (Year)** | **Summary** | **Attributes or scenario relating to healthcare delivery/patient experiences stemming from healthcare delivery #** |
| --- | --- | --- |
| Willingness to Pay (WTP) | | |
| Barner JC & Branvold A (2005) “Patients’ willingness to pay for pharmacist-provided menopause and hormone replacement therapy consultations” Research in Social & Administrative Pharmacy 1: 77-100 | WTP of women for pharmacist-provided menopause and hormone replacement therapy consultations. | WTP linked to patient satisfaction with the services provided by their physician and confidence in pharmacists’ abilities. |
| Bergmo TS & Wangberg, SC (2007) “Patients’ willingness to pay for electronic communication with their general practitioner” Eur J Health Econ, 8: 105-110 | Patient’s WTP for electronic communication with their GP. | Electronic communication with GP. |
| Bishai DM & Lang HC (2000) “The willingness to pay for wait reduction: the disutility of queues for cataract surgery in  Canada, Denmark, and Spain” Journal of Health Economics, 19: 219-230 | Estimates demand curves for a one month reduction in waiting time for cataract surgery. | “Would you be willing to pay {Bid, B} to reduce your waiting time for cataract surgery to less than one month?” |
| Boonen A, Severens, JL, et al (2005) “Willingness of patients with ankylosing spondylitis to pay for inpatient treatment is influenced by the treatment  environment and expectations of improvement” Ann Rheum Dis 64: 1650-1652 | WTP for treatment in a spa resort of patients with ankylosing spondylitis and if the experience of a spa influences the WTP. | Rehabilitation in a local rehabilitation hospital versus a spa resort. |
| Borisova NN & Goodman AC (2003) “Measuring the value of time for methadone maintenance clients: willingness to pay, willingness to accept, and the wage rate” Health Econ 12: 323-334 | WTP to reduce travelling time to collect methadone.  WTA to forgo this reduction. | “If it took you twice as long as usual to travel to this clinic and if you had to pay, what is the MOST money you would be willing to pay for each visit?” |
| Borisova NN & Goodman AC (2004) “The effects of time and money prices on treatment attendance for  methadone maintenance clients” J of Substance Abuse Treatment 26: 43-50 | Identified economic barriers to regular treatment attendance by methadone users. | “If it took you twice as long as usual to travel to this clinic and if you had to pay, what is the MOST money you would be willing to pay for each visit?” |
| Bradford D, Kleit A, et al (2004) “Willingness to pay for telemedicine assessed by the double-bounded dichotomous choice method” Journal of Telemedicine and Health 10: 325-330. | WTP of patients with chronic heart failure for medical care via telemedicine, as an alternative to visits to a physician’s office. | “Would you be willing to pay $20 extra (out of your pocket, in addition to any co-payment you may currently pay) for the opportunity to be examined by your same doctor in your house or apartment by telemedicine, rather than go to [clinic name] for the visit?” |
| Bradford D, Kleit A (2005) “Comparing Willingness to Pay for Telemedicine Across a Chronic Heart Failure and Hypertension Population” Telemedicine and eHealth 11: 430-438 | WTP for a new telemedicine technology for patients treated for chronic heart failure. | Telemedicine appointment versus in-person appointment. |
| Clarke PM (2002) “Testing the convergent validity of the contingent valuation and travel cost methods in valuing the benefits of health care” Health Econ 11: 117-127 | WTP for improving access to mammographic screening in rural areas of Australia. | “Would you be prepared to pay [Bid] in higher taxes to have a mobile breast cancer screening unit visit [Town Name]?” |
| Cocosila M, Archer N, et al (2008) “Would People Pay for Text Messaging Health Reminders?” Telemedicine and eHealth 14 | Willingness of participants who received the service to pay for text message reminders to improve adherence to a recommended health regime. | Text message reminders. |
| Cote I, Gregoire JP, et al (2003) “A Pharmacy-Based Health Promotion Programme in Hypertension: Cost-Benefit Analysis” Pharmacoeconomics 21: 415-428 | Costs and benefits of a pharmacy-based health promotion programme. | “Suppose that the pharmacist can improve your quality of life by taking your blood pressure on a regular basis and by advising you on the best way to take your medication. In addition to what you actually pay for your drug insurance, how much would you be willing to pay per month to benefit from this quality of life improvement?” |
| Delfino M, Holt EW, et al (2008) “Willingness-to-pay stated preferences for 8 health-related quality-of-life domains in psoriasis: a pilot study” J Am Acad Dermatol 59: 439-47. | Pilot test a new method to measure quality of life impact in psoriasis and identify areas of life most affected. | Intimacy, physical comfort, self-care, ability to work or volunteer, ability to concentrate, emotional health, social comfort, ability to sleep. |
| Hong SH, Spadaro D, et al (2005) “Patient valuation of pharmacist services for self care with OTC medications” J Clin Pharm & Therapeutics 30: 193-199 | WTP for pharmacist self-care services on proper use of over-the-counter medications. | Pharmacist self-care service. |
| Leung GM, Yeung RY, et al (2006) “Time costs of waiting, doctor-shopping and private–public sector imbalance: Microdata evidence from Hong Kong” Health Policy 76: 1-12 | Aimed to quantify the disutility of waiting for a specialist consultation. | “What is the maximum amount you are willing to pay to shorten the waiting time for your episode by 2 weeks?” |
| Liang W, Lawrence WF, et al (2003) “Acceptability of diagnostic tests for breast cancer” Breast Cancer Research & Treatment 79: 199-206 | Assessed women’s preferences for having a non-invasive diagnostic test compared to a surgical biopsy. | Non-invasive diagnostic test versus a biopsy. |

| Marra CA, Frighetto L, et al (2005) “Willingness to pay to assess patient preferences for therapy in a Canadian setting” BMC Health Ser Res 5 | To elicit treatment location preferences and WTP from patients referred to an adult outpatient parental antibiotic therapy programme. | Hospital treatment course versus a similar treatment regimen administered in the home. |
| --- | --- | --- |
| Martin-Fernandez J, del Cura-González MI, et al (2010) “Differences between willingness to pay and willingness to accept for visits by a family physician: A contingent valuation study” BMC Public Health 10 | Use of WTP to study the perceptions of patients about the service provided by the family physician. | WTP amount linked to waiting time and relationship with the family physician. |
| Martín-Fernándeza J, Gómez-Gascón T, et al (2010) “Perception of the economic value of primary care services: A willingness to pay study” Health Policy 94: 266-272 | Use of WTP to study the perceptions of patients about the service provided by the family physician. | WTP amount linked to waiting time and relationship with the family physician. |
| Protiere C, Donaldson C, et al (2004) “The impact of information on non-health attributes on willingness to pay for multiple health care programmes” Soc Sci Med 58: 1257-1269 | Explore the impact of the inclusion of non-health attributes on WTP. | First group: “After the intervention, patients would spend 3 days in an intensive care unit and then 10 days in conventional hospitalisation”. Second group, patients could choose whether or not to be in a single room during the conventional hospitalisation period. |
| Qureshi AA, Brandling-Bennett HA, et al (2006) “Willingness-to-Pay Stated Preferences for Telemedicine Versus In-Person Visits in Patients with a History of Psoriasis or Melanoma” Telemedicine and eHealth 12 | WTP for telemedicine versus in-patient clinic visits in patients with a history of psoriasis or melanoma. | Time until inpatient visit; time until telemedicine response. |
| Raza S, Rosen MP, et al (2001) “Patient Expectations and Costs of Immediate Reporting of Screening Mammography” AJR 177: 579-583 | To determine whether patients prefer immediate or delayed results of screening mammography. | Waiting an additional 30–60 min to obtain immediate results. |
| Taylor S, Hourihan F (2009) “Measuring consumer preference for models of diabetes care delivered by pharmacists” Pharmacy Practice 7: 195-204. | Measuring patient preferences for diabetes care provided by a pharmacist. | Information about medication; changes in medication suggested to doctor; information about diet; information about exercise; measurement of blood glucose; management of my disease and any problems. |
| Wan-kin Chan F, Hoi Fan A, et al (2009) “Waiting Time for Cataract Surgery and Its Influence on Patient Attitudes” IOVS 50: 3636-3642 | WTP for private sector cataract surgery. | Reduced waiting time. |

| Time Trade-Off | | |
| --- | --- | --- |
| Salkeld G, Quine S, et al (2004) “What constitutes success in preventative health care? A Case study in assessing the benefits of hip protectors” Social Science & Medicine 59: 1593-1601 | To examine the presence of process utility using the case study of hip protectors. | Feelings or experiences of reassurance, elation, discomfort, anxiety, regret and anguish. |
| Standard Gamble | | |
| Birch S, Melnikow J, et al (2003) “Conservative versus aggressive follow up of mildly abnormal Pap smears: Testing for process utility” Health Economics 12: 879-884 | Testing for process utility in the case of pap smears. | Different management strategies following a low grade abnormal Pap smear. |
| Robinson A, Thomson R, et al (2001) “How patients with atrial fibrillation value different health outcomes: a standard gamble study” J Health Serv Res Policy 6: 92-98 | Aimed to elicit patient valuations of health states relevant to the assessment and prevention of stroke. | GP versus hospital-managed warfarin treatment |
| Multiple valuation methods | | |
| Lehmann HP, Fleisher LA, et al (1999) “Patient Preferences for Early Discharge After Laparoscopic Cholecystectomy” Anesth Analg 88:1280–5 | Patients’ attitudes toward postoperative care at home or in the hospital after laparoscopic  Cholecystectomy.  WTP; Rating Scale and Standard Gamble | Postoperative care at home versus in the hospital setting. |
| McNamee P, Glendinning S, et al (2004) “Chained time trade-off and standard gamble methods: Applications in oesophageal cancer” Eur J Health Econ 5: 81-86 | Chained TTO and SG to value patient preferences over different treatments in the case of oesophageal cancer. | Level of activity (work and interests); relations with family and friends; healthy lifestyle; feeling optimistic about the future. |
| McNamee P & Seymour J (2008) “Incorporation of process preferences within the QALY Framework: A study of alternative methods” Medical Decision Making 28: 433-452 | Using SG & TTO as an alternative method of measuring the benefits of antenatal screening | Whether the person’s most preferred treatment method was received. |
| Ryan M & Watson V (2009) “Comparing welfare estimates from payment card contingent valuation and discrete choice experiments” Health Economics 18: 389-401 | Comparing welfare estimates from payment card contingent valuation and DCE. | Place of screening; type of screening; support of a trained health-care advisor when you receive your test results. |
| Best-Worst Scaling | | |
| Coast J, Flynn TF, et al (2008) “Valuing the ICECAP capability index for older people” Soc Sci Med 67: 874-882 | Valuation of the ICECAP capability index for older people. | Attachment (love & friendship); security (thinking about the future without concern); role (doing things which make you feel valued); Enjoyment; Control. |
| Coast J, Salisbury C, (2006) “Preferences for aspects of a dermatology consultation” Epidemiology and Health Services Research 155: 387-392. | Preferences for aspects of a dermatology consultation. | Waiting time; difficulty/convenience of getting to the appointment; expertise of staff; thoroughness of the consultation. |
| Flynn, TN, Louviere, JJ, et al (2008) “Estimating preferences for a dermatology consultation using Best-Worst Scaling: Comparison of various methods of analysis” BMC Medical Research Methodology 8 | Methodological paper, drawing upon the use of BWS to elicit values for a dermatology consultation.. | Waiting time; difficulty/convenience of getting to the appointment; expertise of staff; thoroughness of the consultation. |
| Swancutt D, Greenfield SM, et al (2008) “Women’s colposcopy experience and preferences: a mixed methods study” BMC Women’s Health 8 | Two stage mixed-methods project to identify experiences of colpscopy and then value these. | The attitude of staff; delays and waiting time; viewing the monitor present in the consultation room; provision of information before the appointment; gender of the colposcopist; the feeling of being rushed. |
| Discrete Choice Experiment & Conjoint Analysis | | |
| Albada A & Triemstra M (2009) “Patients’ priorities for ambulatory hospital care centres. A survey and discrete choice experiment among elderly and chronically ill patients of a Dutch hospital” Health Expectations 12: 92-105 | Patients’ preferences regarding facilities in a centre for ambulatory hospital care. | Consultations with same specialist vs. different specialist; Consecutive consultations vs. consultations on different days; Consultations at the same hospital location vs. at different hospital locations; Time in waiting room less than 15 minutes vs. 15 to 30 minutes; Waiting list shorter than 2 weeks vs. 2 to 6 weeks; Information desk for questions on health and diseases vs. no such desk. |
| Bech M, Gyrd-Hansen D, et al (2007) “Graded pairs comparison- Does strength of preference matter? Analysis of preferences for specialised nurse home visits for pain management” Health Econ 16: 513-529 | Elicited preferences for home visits by specially trained pain nurses from a multidisciplinary pain centre. | Provider; number of consultations per year. |
| Caldow J, Bond C, et al (2006) “Treatment of minor illness in primary care: a national survey of patient satisfaction, attitudes and preferences regarding a wider nursing role” Health Expectations 10: 30-45 | Investigated patient opinion about the provision of nurse-led vs. doctor-led primary health care. | Who you see; waiting time till appointment; length of consultation; continuity of health professional. |
| Cheraghi-Sohi S, Bower P, et al (2007) “Making sense of patient priorities: applying discrete choice methods in primary care using ‘think aloud’ technique” Family Practice doi:10.1093/fampra/cmm007 | Evaluating patient priorities in primary care. | Waiting time; choice of appointment times; attitude of doctor (e.g. warm and friendly); doctor knows you; thoroughness of examination. |

| Clark M, Moro D, et al (2009) “Balancing patient preferences and clinical needs: Community versus hospital based care for patients with suspected DVT” Health Policy 90: 313-319 | Patients’ preferences and willingness to pay (WTP) for different service models for suspected deep vein thrombosis (DVT). | Speed of diagnosis; access; continuity of care; minimizing hospital visits. |
| --- | --- | --- |
| Cunningham MA, Gaeth GJ, et al (1999) “Using choice-based conjoint to determine the relative importance of dental benefit plan attributes” J Dent Educ 65: 391-399 | Explored the importance of specific dental plan features. | Clinic hours of operation. |
| van Dam L, Hol L, et al (2010) “What determines individuals’ preferences for colorectal cancer screening programmes? A discrete choice experiment” 46: 150-159 | A discrete choice experiment was conducted among subjects in the age group of 50–75 years, including both screening-naive subjects and participants of a CRC screening programme. | Location. |
| Fiebig DG, Haas M, et al (2009) “Decisions about Pap tests: What influences women and providers?” Soc Sci Med 68: 1766-1774 | Investigate women’s choices in relation to cervical screening. | GP is your regular GP (or not); GP is male/female. |
| Gerard K, Shanahan M, et al (2003) “Using stated preference discrete choice modelling to inform health care decision-making: A pilot study of breast screening participation” Applied Economics 35: 1073-1085 | Explores the feasibility of applying stated preference discrete choice modelling for use in developing breast screening participation enhancement strategies. | Method of inviting women for screening; information included with invitation; time to wait for an appointment; choice of appointment times; time spent travelling; how staff relate to you; attention paid to privacy; results notification time. |
| Gerard K, Lattimer, V, et al (2004) “Reviewing emergency care systems 2: measuring patient preferences using a discrete choice experiment” Emerg Med J 21: 692-697 | Preferences of patients for emergency services available during usual GP surgery hours. | Initial contact (face-to-face or telephone); advice from doctor, Paramedic or nurse; advised at home/ NHS facility; waiting time; informed of expected wait; quality of contact (time & interruptions). |
| Gerard K & Lattimer, V (2004) “Preferences of patients for emergency services available during usual GP surgery hours: a discrete choice experiment” Family Practice doi:10.1093/fampra/cmh623 | Preferences of patients for emergency services available during usual GP surgery hours. | Initial contact (face-to-face or telephone); advice from doctor, Paramedic or nurse; advised at home/ NHS facility; waiting time; informed of expected wait; quality of contact (time & interruptions). |
| Gerard K, Lattimer V, et al (2006) “The introduction of integrated out-of-hours arrangements in England: a discrete choice experiment of public preferences for alternative models of care” Health Expectations 9: 60-69 | Establishes which generic attributes of general practice out-of-hours health services are important to the public. | Time it takes to make initial contact; time waiting for advice or treatment; informed of expected waiting times; type of contact; professional person providing initial advice or treatment. |

| Gerard K, Salisbury C, et al (2008) “Is fast access to general practice all that should matter? A discrete choice experiment of patients’ preferences” J of Health Serv Res & Pol 13 Suppl 2: 3-10 | Determines the relative importance of factors that influence patient choice in the booking of general practice appointments. | Day of appointment; professional person (nurse/doctor); time of day of appointment; length of appointment. |
| --- | --- | --- |
| Gidman W, Elliott R, et al (2007) “A comparison of parents and pediatric anesthesiologists’ preferences for attributes of child daycase surgery: a discrete choice experiment” Pediatric Anesthesia 17: 1043-1052 | Aimed to elicit and compare the relative importance of attributes of pediatric daycase surgery provision to parents and anaesthesiologists. | Parental presence at the induction of anesthesia. |
| Griffith GL, Tudor Edwards R, et al (2008) “Patient preferences and National Health Service costs: a cost-consequence analysis of cancer genetic services” Familial Cancer 8: 265-275 | Aimed to elicit patients’ willingness to pay for cancer genetic services. | Staff seen for counselling; waiting time for letter confirming risk status; distance to counselling; duration of counselling; availability of genetics testing. |
| Haas M (2005) “The impact of non-health attributes of care on patients’ choice of GP” Australian J of Primary Health 11: 40-46 | The impact of non-health attributes of care on patients’ choice of GP. | The doctor treats you with dignity; the doctor recognises your pain/stress; the doctor is trustworthy; the doctor takes notice of what you say or do about your health; the doctor reassures you; the doctor gives you information; the doctor accepts your decisions. |
| Hall J, Fiebig GD, et al (2006) “What influences participation in genetic carrier testing? Results from a discrete choice experiment” J of Health Economics 25: 520-537 | Explores factors that influence participation in genetic testing programs. | Whether you are told your carrier status as an individual or whether you are told your risk as a couple; where you go to be tested; time waiting for results. |
| Haughney J, Fletcher M, et al (2007) “Features of asthma management: quantifying the patient Perspective” BMC Pulmonary Medicine 7 | Quantifies the importance of different features of asthma management from the patient perspective. | Use of a written personalised asthma action plan; where you attend for asthma crisis management; Whether you’re encouraged to speak to a doctor/nurse before changing your own therapy. |
| Hjelmgren J & Anell A (2007) “Population preferences and choice of primary care models: a discrete choice experiment in Sweden” Health Policy 83: 314-322 | Examined which attributes are important when individuals choose between primary care models. | Registered with GP/primary care team; patient influence; patient choice; user charges; waiting time for non-emergency visit. |

| Hole AR (2008) “Modelling heterogeneity in patients’ preferences for the attributes of a general practitioner appointment” J of Health Econ 27: 1078-1094 | Examines preferences relating to general practitioner appointments. | Number of days wait for an appointment; cost of appointment; flexibility of appointment times; Doctor’s interpersonal manner; Doctor’s knowledge of the patient; thoroughness of physical examination. |
| --- | --- | --- |
| Hundley V, Ryan M, et al (2001) “Assessing women’s preferences for intrapartum care” Birth 28 | Use of DCE to assess the importance to women of different aspects of intrapartum care. | Continuity of contact with the midwife; appearance of the room; your involvement in decisions. |
| Jan S, Mooney G, et al (2000) “The use of conjoint analysis to elicit community preferences in public health research: a case study of hospital services in South Australia” Australian & New Zealand J of Public Health 24: 64-70 | Valued attributes found to influence the choice of hospital services. | Travel time to hospital; access to parking and public transport; average waiting time for elective surgery; average waiting time in casualty. |
| Johnson FR, Ozdemir S, et al (2010) “Effects of simplifying choice tasks on estimates of taste heterogeneity in stated-choice surveys” Social Science & Medicine 70: 183-190 | DCE as part of a study examining preferences for HIV testing methods. | Location; timeliness/accuracy; privacy; availability of counselling. |
| Kimman ML, Dellaert B, et al (2010) “Follow-up after treatment for breast cancer: one strategy fits all? An investigation of patient preferences using a discrete choice experiment” Acta Oncologica 49: 328-337 | Explored patients’ preferences for follow-up after breast cancer. | Attendance at educational group meetings; frequency of visits; waiting time in minutes; contact face-to-face or by telephone; healthcare provider (e.g. breast care nurse/GP). |
| Longo MF, Cohen DR, et al (2006) “Involving patients in primary care consultations: assessing preferences using discrete choice experiments” British Journal of General Practice 56: 35-42 | Identifies patient preferences for shared decision making in primary care. | Doctor listens; Amount of information about your health problem and its treatment; How easily the information is understood; who chooses treatment; length of consultation. |
| Maddala T, Phillips KA, et al (2003) “An experiment on simplifying conjoint analysis designs for measuring preferences” Health Econ 12: 1035-1047 | Methodological paper which reports the elicitation of preferences relating to HIV testing. | Location of test; cost; test format/administration; waiting time for test results and accuracy; privacy; sources of information. |
| Michaels J, Brazier J, et al (2000) “Cost and outcome implications of the organisation of vascular services” Health Technology Assessment 4 (11) | Describes an application of conjoint analysis to the provision of vascular surgery with a view to establishing the tradeoffs vascular patients are prepared to make with respect to a number of key attributes of service organisation. | Months between diagnosis and operation; local or non-local treatment; length of stay; whether you see the same or different staff; local or non-local follow-up services. |
| Miguel FS, Ryan M, et al (2005) “Irrational stated preferences: a quantitative and qualitative investigation” Health Econ 14: 307-322. | Nurse led care in primary care. | Who you see; waiting time; length of consultation; continuity of health professional. |
| Nieboer AP, Koolman X, et al (2010) “Preferences for long term care services: Willingness to pay estimates derived from a discrete choice experiment” Soc Sci Med 70: 1317-1325 | Relative preferences of over 50s for long-term care and willingness to pay for these. | Number of hours care per week; organised social activities; transportation service; living situation; who provides care; standardised versus individually determined content of care; coordination of care delivery; punctuality; waiting list in months. |
| Peacock S, Apicella C, et al (2006) “A discrete choice experiment of preferences for genetic counselling among Jewish women seeking cancer genetics services” British Journal of Cancer 95: 1448-1453 | Which aspects of breast cancer genetic counselling are important? | Genetic counselling, gene and risk information; giving advice about cancer surveillance; preparing for genetic testing (preparation); and, assistance with decision-making. |
| van der Pol M & McKenzie L (2009) “Costs and benefits of tele-endoscopy clinics in a remote location” J of Telemedicine and Telecare DOI:10.1258/jtt.2009.090609 | Elicits patients’ preferences over how care is delivered. | Drive time; waiting time. |
| Pitchforth E, Watson V, et al (2008) “Models of intrapartum care and women’s trade-offs in remote and rural Scotland: a mixed methods study” BJOG 1: 560-569 | A DCE questionnaire explored women’s preferences for and trade-offs between these attributes. | Staff involved (midwife or consultant led); time travelled to delivery unit. |
| Porteous T, Ryan M, et al (2007) “Preferences for self-care or professional advice for minor illness: a discrete choice experiment” British Journal of General Practice 57 911-917 | To determine the relative importance of factors that influence decision making in the management of minor illness. | Type of management (GP/ practice nurse/ pharmacy/ complementary/ NHS24/ Self-care/ do nothing); availability. |
| Ratcliffe J, Van Haselen R, et al (2002) “Assessing patients’ preferences for characteristics associated with homeopathic and conventional treatment of asthma: a conjoint analysis study” Thorax 57: 503-508. | Investigates the preferences of patients with asthma for attributes or characteristics associated with treatment for their asthma. | Extent to which the doctor gives sufficient time to listen to what the patient has to say and discuss treatment options; Extent to which the patient sees the same doctor for every visit; extent to which the doctor treats the patient as a whole person; travel. |
| Richardson G, Bojke, C, et al (2009) “What outcomes are important to patients with long-term conditions? A discrete choice experiment” Value in Health 2: 331-339 | Valued outcomes important to patients with long-term conditions. | Self-efficacy (confidence in their ability to manage their own condition); and access to GPs; compared relative to health outcomes. |
| Roux L, Ubach C, et al (2004) “Valuing the benefits of weight loss programs: An application of the discrete choice experiment” Obesity Research 12: 1342-1351 | Explored the relative importance of program attributes in a sample of 165 overweight adults enrolled in community weight loss programs. | Travel time required to attend; extent of physician involvement (e.g., none, monthly, every 2 weeks); components (e.g. diet, exercise, behaviour change) emphasized; focus (e.g. group, individual). |
| Rubin G, Bate A, et al (2006) “Preferences for access to the GP: a discrete choice experiment” British Journal of General Practice 56: 743-748 | Investigates patient preferences when making a routine appointment for a GP. | Time to appointment; choice of doctor; choice of time. |
| Ryan M (1999) “Using conjoint analysis to take account of patient preferences and go beyond health outcomes: an application to in vitro fertilisation” Soc Sci Med 48: 535-546 | Health, non-health and process attributes associated with in vitro fertilisation. | Attitudes of staff toward you; continuity of contact with same staff; time on waiting list for IVF attempt; follow-up support. |
| Ryan M, Bate, A, et al (2001) “Use of discrete choice experiments to elicit preferences” Quality in Health Care 10: i55-i60. | A study of the preferences of patients attending the rheumatology outpatient clinic. | The medical staff you see; time in waiting area; continuity of contact with same staff; phone-in/advice line service; length of consultation. |
| Ryan M, Diack J, et al (2005) “Rapid prenatal diagnostic testing for Down Syndrome only or longer wait for full karyotype: the views of pregnant women” Prenatal Diagnosis 25: 1206-1211 | Views of pregnant women on rapid prenatal testing for Down Syndrome. | Level of information; wait for results. |
| Ryan M, McIntosh E, et al (2000) “Trade-offs between location and waiting times in the provision of health care: the case of elective surgery on the Isle of Wight” J of Public Health Medicine 22: 202-210 | Elicited community views on the importance of reducing waiting times. | Operation waiting time; travel cost. |
| Ryan M, Major K, et al (2005) “Using discrete choice experiments to go beyond clinical outcomes when evaluating clinical practice” Journal of Evaluation in Clinical Practice 11: 328-338 | To estimate the monetary value of reducing waiting time, as well as changes in duration of appointment in the case of rheumatology services. | Length of wait; Time with doctor; Pain management service. |
| Ryan M, Netten A, et al (2006) “Using discrete choice experiments to estimate a preference-based measure of outcome – an application to social care for older people | Estimation of quality weights for a social care outcome measure | Food and nutrition; Personal care; Safety; social participation and involvement; control over daily living. |
| Ryan M & Wordsworth S (2000) “Sensitivity of Willingness to Pay Estimates to the Level of Attributes” Scottish Journal of Political Economy 47: 504-524 | Methodological study using smear testing as a case study. | Time for results. |
| Salkeld G, Solomon M, et al (2005) “Discrete-choice experiment to measure patient preferences for the surgical management of colorectal cancer” British Journal of Surgery 92: 742-747 | Assessed the relative importance of the ‘attributes of trust’ between surgeon and patient with colorectal cancer. | Surgeon has additional training; type of hospital in which you are treated; surgeon’s words and explanations are easy/difficult to understand; who decides treatment. |

| Schwappach D & Strasmann TJ (2007) “Does location matter? A study of the public’s preferences for surgical care provision” J of Evaluation in Clinical Practice 13: 259-264 | A study of the public’s preferences for surgical care provision. | Location of care; waiting time; travel time; specialised/experienced provider; staff continuity. |
| --- | --- | --- |
| Scott A, Watson MS, et al (2003) “Eliciting preferences of the community for out of hours care provided by general practitioners: a stated preference discrete choice experiment” Soc Sci Med 56: 803-814 | Elicited the preferences of users and non-users (i.e. the community) for different models of out of hours care. | Where your child is seen; who your child sees; time taken between the telephone call and treatment being received; whether the doctor seems to listen to what you have to say. |
| Seston EM, Elliott RA, et al (2007) “Women’s preferences for the provision of emergency hormonal contraception services” Pharm World Sci 29: 183-189 | Elicit women’s preferences for routes of supply for emergency hormonal contraception. | Number of hours service is open; Medical staff seen; Wait to be seen at service; Privacy of consultation; Attitude of staff. |
| Shackley P, Slack R, et al (2001) “Vascular patients’ preferences for local treatment: an application of conjoint analysis” J of Health Serv Res & Pol 6: 151-157 | Investigation of the extent to which vascular patients are willing to trade expected health outcomes for improvements in non-health benefits. | Months between diagnosis and operation; local or non-local treatment; length of stay; whether you see the same or different staff; local or non-local follow-up services. |
| Taylor S & Armour C (2002) “Acceptability of willingness to pay techniques to consumers” Health Expectations 5: 341-356 | WTP of pregnant women attending an antenatal clinic for a preferred treatment. | Place of care. |
| Turner D, Tarrant C, et al (2007) “Do patients value continuity of care in general practice? An investigation using stated preference discrete choice experiments” 12: 132-137 | Estimates the relative importance to patients of continuity of care compared with other aspects of a primary care consultation. | Staff seen; staff you do not know/know and trust; who does/ does not have information about your full medical history; waiting time. |
| Walzer S (2007) “What do parents want from their child’s asthma treatment?” Therapeutics and Clinical Risk Management 3: 167-175 | Assessed the relative importance of attributes of asthma treatment. | Provision of information about long-term effects |
| Watson V, Ryan M, et al (2009) “Valuing experience factors in the provision of Chlamydia screening: an application to women attending the family planning clinic” Value in Health 12: 621-623 | Examined women’s preferences for characteristics of Chlamydia screening. | Place of screening; support of trained health-care advisor. |
| Kinghorn (2010) “Developing a capability approach to measure and value quality of life: an application to chronic pain” PhD Thesis | Use of a Swing-Weighting method to value capabilities identified as relevant to patients with chronic pain. | Self-respect; Enjoyment; Physical & mental well-being; relationships; identity; parenting; social interaction; independence & control; remaining physically & mentally active. |

# Where the exact scenario is given in the paper, this is reproduced in the table, otherwise those attributes relating to healthcare delivery/experiences stemming from healthcare delivery are summarised.
